# Isolation and Identification of a Natural Reassortant Mammalian Orthoreovirus from Least Horseshoe Bat in China

**DOI:** 10.1371/journal.pone.0118598

**Published:** 2015-03-17

**Authors:** Lihua Wang, Shihong Fu, Lei Cao, Wenwen Lei, Yuxi Cao, Jingdong Song, Qing Tang, Hailin Zhang, Yun Feng, Weihong Yang, Guodong Liang

**Affiliations:** 1 State Key Laboratory for Infectious Disease Prevention and Control, Key Laboratory for Medical Virology, National Institute for Viral Disease Control and Prevention, Chinese Center for Disease Control and Prevention, Beijing, China; 2 Collaborative Innovation Center for Diagnosis and Treatment of Infectious Diseases, Hangzhou, China; 3 Yunnan Institute of Endemic Diseases Control and Prevention, Yunnan, China; Linneaus University, SWEDEN

## Abstract

**Background:**

Mammalian orthoreoviruses (MRVs) have a wide geographic distribution and can infect virtually all mammals. Infections in humans may be either symptomatic or asymptomatic. This study describes the isolation and identification of a natural reassortant MRV from least horseshoe bats (*Rhinolophus pusillu*) in China, referred to as RpMRV-YN2012.

**Methods and Results:**

The RpMRV-YN2012 was obtained from urine samples of *Rhinolophus pusillus* by cell culture. Negative-staining electron microscopy revealed that RpMRV-YN2012 was a non-enveloped icosahedral virus with ∼75 nm in diameter. Polyacrylamide gel electrophoresis (PAGE) migration patterns of the genome segments showed that RpMRV-YN2012 contained 10 segments in a 3:3:4 arrangement. The whole genome sequence of RpMRV2012 was determined. The consensus terminal sequences of all segments of 5’-GCUAh…yUCAUC-3’ (h = A, U or C; y = C or U) were similar to the MRV species within the genus *Orthoreovirus*. Its evolution and evidence of genetic reassortment were analyzed by sequence comparison and phylogenetic analysis. The results showed that RpMRV-YN2012 is a novel serotype 2 MRV that may have originated from reassortment among bat, human, and/or pig MRV strains which associated with diarrhea, acute gastroenteritis and necrotizing encephalopathy in animals and humans.

**Conclusions:**

RpMRV-YN2012 is a novel bat reassortant MRV, which may have resulted from a reassortment involving MRVs known to infect humans and animals. It is necessary to identify whether RpMRV-YN2012 is associated with diarrhea, acute gastroenteritis and necrotizing encephalopathy in clinical patients. In addition, we should carefully monitor its evolution and virulence in real time.

## Introduction

Mammalian orthoreoviruses (MRVs), prototypes of the genus *Orthoreovirus* (family *Reoviridae*), are non-enveloped viruses with a segmented double-stranded RNA (dsRNA) genome (∼23,500 bp) [1]. MRVs have four major serotypes (type 1 Lang, type 2 Jones, type 3 Dearing, and type 4 Ndelle) [1, 2]. Each MRV particle contains 10 genome segments divided into three size classes based upon their characteristic mobility during gel electrophoresis: three large (L1, L2 and L3) segments, three medium segments (M1, M2 and M3), and four small segments (S1, S2, S3 and S4) [1–3]. The virions have an average diameter of 70–80 nm with a typical icosahedral, double-layered protein capsid structure [1–3].

Although MRVs had been assumed to cause mild respiratory or gastrointestinal diseases, recent studies have shown that they can cause severe illnesses in humans and other mammals, including upper respiratory tract infections, encephalitis, and diarrhea [4, 5]. MRVs have been isolated from many mammalian species, including humans and bats [4, 5]; however, the natural reservoirs or direct progenitors remain unclear. The significance of bats as a source of emerging infectious diseases has been recognized. Bats also are being increasingly recognized as reservoir hosts for viruses which can cross species to infect humans and other domestic and wild mammals [6–8]. Indeed, many recent outbreaks of emerging viruses, such as the Hendra virus, Nipah virus, Ebola virus and severe acute respiratory syndrome coronavirus (SARS-like CoVs), have been associated with bat transmission events [9–13].

Here, we describe the first isolation of a novel natural reassortant MRV strain, named RpMRV-YN2012, from the least horseshoe bat (*Rhinolophus pusillus*) in China. The whole genome sequence of RpMRV2012 was determined. Its evolution and evidence of genetic reassortment were analyzed by sequence comparison and phylogenetic analysis.

## Materials and Methods

### Ethics Statement

Bats were treated according to the guidelines of Regulations for the Administration of Laboratory Animals (Decree No. 2 of the State Science and Technology Commission of the People's Republic of China, 1988). The sampling was approved by the Ethics Committee of Institute for Viral Disease Control and Prevention, Chinese Center for Disease Control and Prevention.

### Sample collection

Clean plastic sheets measuring 2.0 by 2.0m were placed under bat roosting sites at approximately 17:00. The fresh urine and fecal samples were collected at the following morning (approximately 7:30 to 8:00). Pharyngeal swab and anal swab samples from captured bats were immersed into maintenance media in a virus sampling tube (Yocon, China). After collecting the swab samples, all bats were released at their capture site. The samples were transported to the laboratory under chilled conditions and stored at −80°C until being processed.

### Cell culture and virus isolation

Cell lines used in this study were BHK-21(ATCC CCL-10) and Tb1Lu (ATCC CCL88). Cells were grown in Dulbeco’s Modified Eagle Medium (DMEM) containing high glucose (Invitrogen, Breda, The Netherlands), supplemented with penicillin, streptomycin and 10% fetal bovine serum (FBS) (Invitrogen, Breda, The Netherlands) at 37°C in the presence of 5% CO_2_.

The urine and fecal samples were thawed at 4°C and centrifuged at 16,000xg for 5 min to pellet debris. Supernatant was filtered through a 0.45-μm filter (Millipore) to remove bacterium-sized particles, and then was diluted 1:10 in cell culture media. Two aliquots of 200 μl diluted supernatant were added to monolayer BHK-21and Tb1Lu cells in a 24-well plate separately. After rocked for 2 h at 37°C, 1ml of fresh cell culture media was added and then incubated for 7 days at 37°C. The flasks were observed daily for toxicity, contamination, or viral cytopathic effect (CPE). By CPE occurrence in the third subcultivation, the supernatant was passed to an 80–90% confluent 175 cm^2^ flask of fresh cells and incubated at cultivation conditions for four days. To harvest virus particles, cells were homogenized by three freeze-thaw cycles and the resulting suspension was purified from cell debris by low-speed centrifugation. Aliquots were used as viral stocks and stored at −80°C.

### Virus cloning

Viral supernatants were applied to six-well plates (Corning, USA) of confluent BHK-21 cells with serial dilution and incubated for one hour. Plates were first overlaid with medium containing 75% agarose and then with medium containing neutral red vital stain after three days incubation at 37°C in a 5% CO_2_ incubator. Plaques of different sizes and shape were shattered in 500 ul MEM medium after being picked out using a sterile pipette tip. As described previously [14], this process was repeated until a single plaque shaped virus was obtained.

### Electron microscopy

When 80% BHK-21 cells showed CPE, the supernatant of the medium containing viral particles were concentrated at 40,000 rpm for 25 min in a Hitachi centrifuge (Hitachi, Japan). For negative staining, one drop of culture supernatant was adsorbed on Formvar carbon coated grid (1 min), stained with 3% phosphotungstic acid (pH 6.3) (1 min), and inactivated with ultraviolet irradiation before examination. The infected cells were physically detached by cell scraper. After 3000xg for 15 min, the cells were fixed 2.5% glutaraldehyde in 0.1 mol/L phosphate buffer (pH 7.4) overnight at 4°C to make ultrathin sections. The viral particles were observed using transmission electron microscopy (TECNAI 12, FEI, Blackwood, NJ) with an acceleration voltage of 80 kV.

### Migration of genome segments

Virion from culture supernatant was harvested and viral RNA was extracted using the QIamp viral RNA kit (Qiagen, Germany). Fifteen ul of viral RNA was run on a 10% SDS polyacrylamide/Bis gel under denaturing and reducing conditions at 150 V for 4 hrs at room temperature. The gel was washed with distilled water, stained by Fast Silver Stain Kit (Beyotime, China) according to the manufacturer's protocol before the photo was taken.

### Complete genome sequencing including 5’- and 3’-untranslated regions

Whole genome sequences of 10 segments (L1–L3, M1–M3, S1–S4) of the isolate were determined by DNase-sequence-independent single primer amplification (DNase-SISPA) and the rapid amplification of cDNA ends (RACE) methods as described previously [15]. The genome sequences of all segments were reconfirmed by overlapping nested PCRs with specific primers (Table 1) derived from the completed viral genomes.

**Table 1:** Primers used for overlapping RT-PCR confirmation of RpMRV-YN2012.

Primer Name	Orientation	Sequence (5'-3')	Position
L1_F1	Sense	GCTACACGTTCCACGACAATGTCATC	1–26
L1_R1	Antisense	GTGCCGATCTAGCATACTTAGTGGAC	966–991
L1_F2	Sense	GCATGTATGAATCTCTAGAAGGAGGG	869–894
L1_R2	Antisense	CTAGCGACACCTTCATGTATAGCC	1830–1853
L1_F3	Sense	CATGTGACGCTAGTATTACCTGGG	1781–1804
L1_R3	Antisense	CATGAATAACGTATCCAACGCTGCC	2522–2546
L1_F4	Sense	GACATAGCGTATGATGGGACTGC	2334–2356
L1_R4	Antisense	CCTAGTACTCTGAAGCATTGGTCC	3304–3327
L1_F5	Sense	GCTTCTCGAAGTTGTTAGAGGCG	3161–3183
L1_R5	Antisense	GATGAGTTGACGCACCACGGCCCATG	3829–3854
L2_F1	Sense	GCTATTGGCGCGATGGCGAACG	1–22
L2_R1	Antisense	TAGTAGCCAGCAGGGACAGC	922–941
L2_F2	Sense	TTGAGATGGGGAGCGCAGTATGTGG	865–890
L2_R2	Antisense	GTAAGCGATGATTGCTGGTGT	1987–2006
L2_F3	Sense	CTTTCCGACCAGAACTGTGTGGCAC	1863–1887
L2_R3	Antisense	CATGTGGTACGGCATATCTTCT	2912–2933
L2_F4	Sense	CGAGATGAGCCGTATTCTGATATGG	2878–2903
L2_R4	Antisense	TGAATTAGGCGCGCTCACGAGGGAC	3889–3913
L3_F1	Sense	GCTAATCGTCAGGATGAAGCGGATT	1–25
L3_R1	Antisense	GCCATGATGACGGATGAATCTC	966–987
L3_F2	Sense	ACCGTTTCCAGAGGCGGCAGTGTCT	895–919
L3_R2	Antisense	AGGCGTTGAGAAAGCACTCG	2037–2056
L3_F3	Sense	CAATATGATGGTCGGTTTTGAAACG	1972–1997
L3_R3	Antisense	CGTCCTGCCATTGTACTGTTG	3018–3037
L3_F4	Sense	TGATCCAAGGATGACGCAATTAGCG	2983–3009
L3_R4	Antisense	TGAATTGGCCCAACTAGCATCGAGC	3876–3899
M1_F1	Sense	GCTATTCGCGGTCATGGCTTACATC	1–25
M1_R1	Antisense	AGTATTGACAATGCGTCCTTCTATC	1101–1124
M1_F2	Sense	AGAAGACGGAATGTTCACAGATTGG	1039–1064
M1_R2	Antisense	GATGAAGCGCGTACGTAGTCTTAGC	2280–2300
M2_F1	Sense	GCTAATCTGCTGACCGTTACTCTGC	1–25
M2_R1	Antisense	CGACACGCGTCCCACCTCTTAGATT	1108–1131
M2_F2	Sense	GGTTGCGGATAACACCGGAACGAAT	1073–1098
M2_R2	Antisense	GATGATTTGCCTGCGTCCCTTAACC	2180–2203
M3_F1	Sense	GCTAAAGTGACCGTGGTCATGGCTT	1–25
M3_R1	Antisense	TCTCATCCATCAATAGGGCACATAG	1226–1249
M3_F2	Sense	TTGTACACCTTGTCTACGCATAATG	1192–1216
M3_R2	Antisense	TGAATGGGGGTCGGGAAGGCTTAAG	2292–2315
S1-F1	Sense	GCTATTCGTACTGATGTCTGAGCTT	1–25
S1-R1	Antisense	GATGAATCGCCGTCGTGCCGGACGG	1414–1437
S2-F1	Sense	GCTATTCGCTGGTCAGTTATGGCTC	1–25
S2-R1	Antisense	GATGAATGTGTGGTCAGTCGTGAAG	1308–1331
S3-F1	Sense	GCTAAAGTCACGCCTGTTGTCGT	1–23
S3-R1	Antisense	GATGATTAAGCGCCACCCACCACC	1176–1198
S4-F1	Sense	GCTATTTTTGCCTCTTCCCAAACGT	1–25
S4-R1	Antisense	GATGAATGAAGCCTGTCCCACGTC	1174–1196

### Sequence analysis and phylogenetic comparisons

The nucleotide (nt) sequences and deduced amino acid (aa) products were analyzed and assembled using the DNASTAR program (Lasergene). BLAST searches were carried out using the NCBI server (www.ncbi.nlm.nih.gov) with all available databases. An ORF search was performed with the ORF Finder of the NCBI. Sequences of other Orthoreoviruses were downloaded from GenBank and used to build the phylogenetic trees. Sequences were aligned using the CLUSTAL_X, version 2.0. Calculations of nucleic acid and amino acid sequence identities were performed using the MEGA v6.06 software with the default settings (www.Megasoftware.net) [16]. Maximum-likelihood trees of of L, M and S segments were also generated in MEGA, using the p-distance and the Poisson correction algorithms. The robustness of the branching was evaluated by bootstrapping using 1,000 replications.

### Prevalence study

The throat and anal swabs were thawed at 4°C and centrifuged at 16,000xg for 5 min to pellet debris. Supernatant was filtered through a 0.45-μm filter (Millipore) to remove bacterium-sized particles, and aliquot of 200 μl used for RNA extraction using the QIAamp Viral RNA Mini kit (Qiagen) according to the manufacturer's protocol. Extracted RNA was converted to double-stranded cDNA with random hexamers. The existence of viral RNA in the collected throat and anal swabs were detected by a reported RT-PCR procedure targeting the conserved regions of the L1 genome segment of MRV was used [17].

## Results

### Isolation and preliminary identification of RpMRV-YN2012

**Fig 1:**
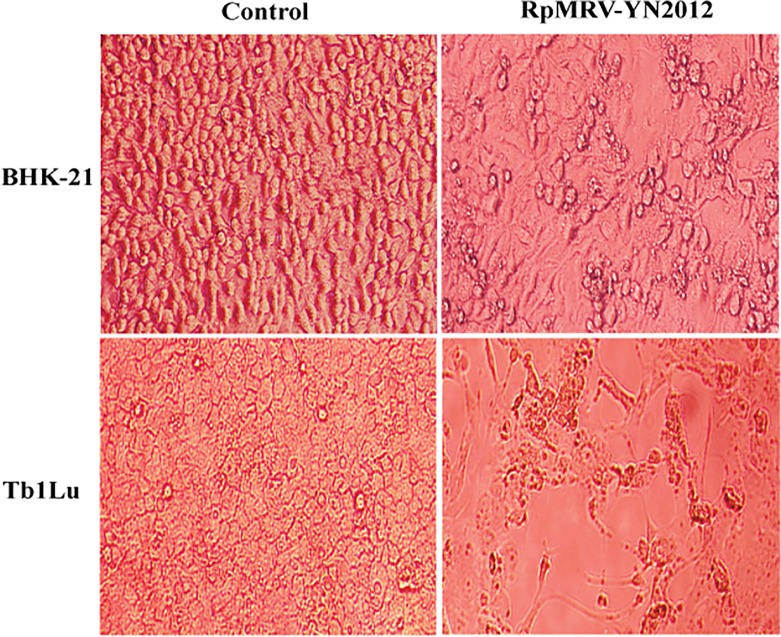
Cytopathic effects of RpMRV-YN2012 on BHK-21 and Tb1Lu cells after three days of infection (200X).

**Fig 2:**
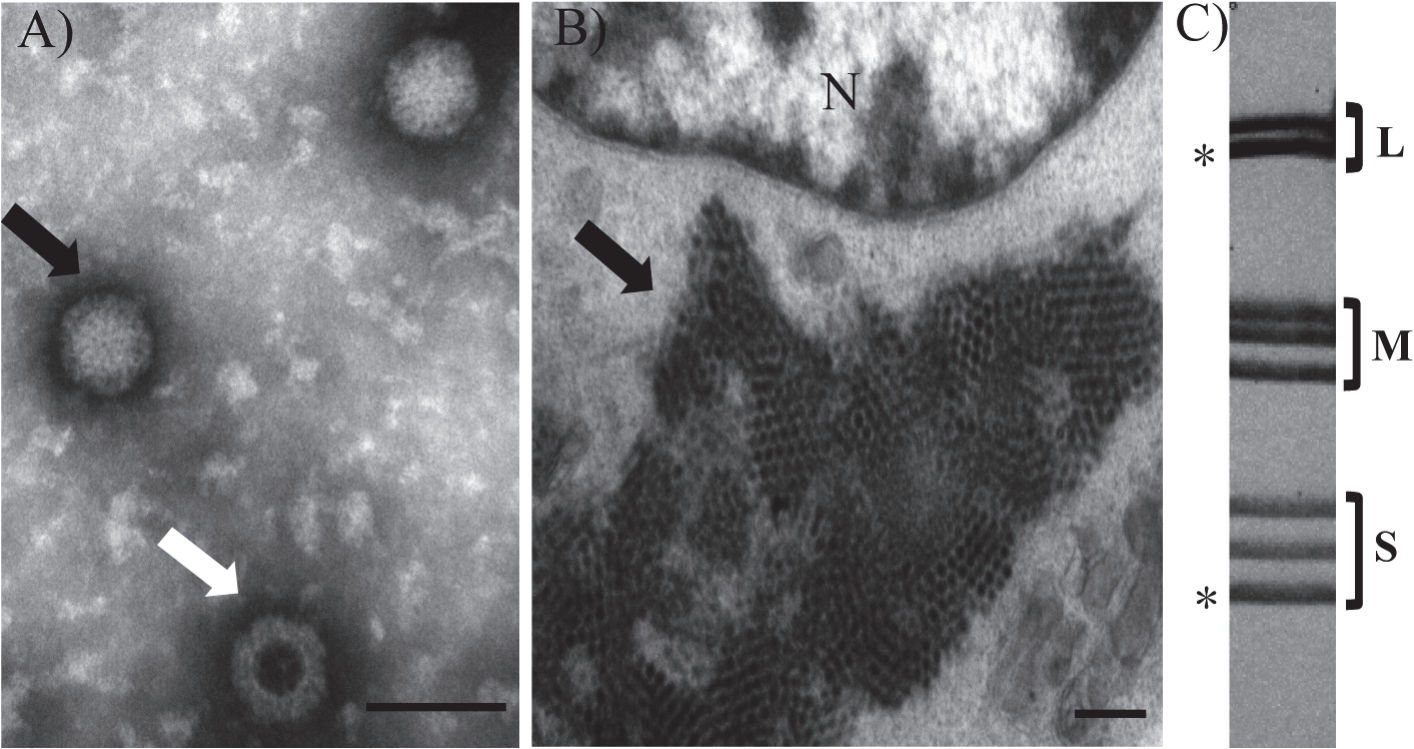
Electron micrographs and genome segment profile of RpMRV-YN2012. **(A)** Negative-stained RpMRV-YN2012. The black arrow indicates an intact particle; the white arrow indicates an empty particle (scale bar: 200 nm). **(B)** Image of an ultrathin section of RpMRV-YN2012-infected BHK-21 cell. N, nucleus; black arrows, paracrystalline viral arrays (scale bar: 0.5 μm). **(C)** Genome segment profile of RpMRV-YN2012. The genome segments were separated on a 10% SDS-polyacrylamide gel. The classes of genome segments (L, M, and S) are labeled on the right. The asterisk (*) indicates co-migrating bands.

Fifteen pool urine samples and fifteen pool feces samples were collected from six bat roosts in southwestern Yunnan Province during July and August, 2012. Roosts were occupied mainly by *Rhinolophus luctus*, *Rhinolophus affinis*, *Rhinolophus pusillus*, and *Myotis daubentonii*. Isolation from pool urine and pool feces samples was attempted in BHK-21 and Tb1Lu cell lines. One isolate was obtained from one pool urine samples of *Rhinolophus pusillus*, designated RpMRV-YN2012. The isolate caused cytopathogenic effects (CPE) 3–4 days post-inoculation in both cell lines. These CPE included granulating, shrinking, rounding, seining, and falling off (Fig. 1). The isolate was plaque-purified and visualized by negative-staining electron microscopy (Fig. 2A), revealing the presence of many non-enveloped icosahedral virus particles of ∼75 nm in diameter, morphologically related to reoviruses. Ultra-thin sections of infected BHK-21 cells displayed typical electron-dense virus particles, organized in a paracrystalline pattern within the cytoplasm (Fig. 2B). Polyacrylamide gel electrophoresis (PAGE) migration patterns of the genome segments showed that RpMRV-YN2012 contained 10 segments in a 3:3:4 arrangement, typical of reoviruses (Fig. 2C).

### Genome organization and characteristics of RpMRV-YN2012

By DNase-SISPA and RACE) methods, the whole genome sequences of 10 segments (L1–L3, M1–M3, S1–S4) of RpMRV-YN2012 were determined. The genome sequences of all segments were reconfirmed by overlapping nested PCRs with specific primers (Table 1) derived from the completed viral genomes and deposited in GenBank (JQ412755–JQ412764).

The complete genome of RpMRV-YN2012 was 23,578 nt in length, including segments L1 to L3, M1 to M3, and S1 to S4 (3,854 nt, 3,915 nt, 3,900 nt, 2,304 nt, 2,203 nt, 2,240 nt, 1,437 nt, 1,331 nt, 1,198 nt, and 1,196 nt, respectively), encoding proteins λ1(1,275 aa), λ2 (1,289 aa), λ3 (1,267 aa), μNS (721aa), μ1 (708 aa), μ2 (736 aa), δ1 (455 aa), δ1s (114 aa), δ2 (418 aa), δNS (366 aa), and δ3 (365 aa) (Table 2). The consensus terminal sequences of all segments of 5’-GCUAh…yUCAUC-3’ (h = A, U or C; y = C or U) (Table 2) were similar to the MRV species within the genus *Orthoreovirus* (8), revealing the MRV character of RpMRV-YN2012.

**Table 2:** Lengths of the coding and untranslated regions of each of the 10 genomic segments of RpMRV-YN2012.

			5′UTR		3′UTR	
Segment	Length (bp)	Protein (aa)	Length (bp)	Terminal sequence	Length (bp)	Terminal sequence
L1	3854	1267	18	5′-GCUAC—	32	-CUCAUC-3′
L2	3915	1289	12	5′-GCUAU—	33	-UUCAUC-3′
L3	3900	1275	13	5′-GCUAA—	59	-UUCAUC-3′
M1	2304	736	13	5′-GCUAU—	80	-UUCAUC-3′
M2	2203	708	29	5′-GCUAA—	47	-AUCAUC-3′
M3	2240	721	18	5′-GCUAA—	56	-CUCAUC-3′
S1	1437	455	13	5′-GCUAU—	57	-UUCAUC-3′
S2	1331	418	18	5′-GCUAU—	56	-UUCAUC-3′
S3	1198	366	27	5′-GCUAA—	70	-AUCAUC-3′
S4	1196	365	32	5′-GCUAU—	66	-UUCAUC-3′

Pair-wise nucleotide (nt) and deduced amino acid (aa) comparisons between RpMRV-YN2012 and other orthoreoviruses, including the prototype MRVs, were performed for all ten segments (Table 3). The results showed that six segments (L1–L3, M2, M3, S1) of RpMRV-YN2012 were closely related to those of human reoviruses (SI-MRV01 and tou05), which isolated from patients with acute gastroenteritis and acute necrotizing encephalopathy recently in Slovenia and France [5, 18]. Interestingly, M1 segment of RpMRV-YN2012 is closest to that of pig diarrhea associated reoviruses (GD-1, SC-A and SHR-A) isolated in China recently [19, 20], and is very distinct from MRVs originated human or other animals. These pig strains have higher sequence identity (94.9–96.2%/96.8–97.5%, nt/aa identity). Identities of nt and aa sequences of M1 between RpMRV-YN2012 and the pig strains ranged from 93.9 to 96.2% and from 96.8 to 97.9%, respectively. The three other segments (S2-S4) shared high sequence similarity with bat-originated MRV strain 342/08, isolated in Germany in 2008 [21] (nt and aa identities from 94.9 to 98.1% and from 98.3 to 99.2%, respectively).

**Table 3:** Nucleotide and amino acid identities for segments of novel bat orthoreovirus RpMRV-YN2012, China.

RpMRV-YN2012	MRV prototype strains				Bat reovirus	Human reoviruses		Pig reoviruses		
	T1L	T2J	T3D	T4N	342/08	SI-MRV01	MRV2tou05	GD-1	SC-A	SHR-A
L1	89.9/98.6	75.2/92.4	90.0/99.0	90.7/97.9	90.3/98.7	**91.2/99.1**	90.0/98.8	90.4/98.2	90.6/98.5	91.1/99.2
L2	76.3/92.5	73.1/80.7	86.6/97.3	NA	86.6/97.7	**86.7/97.8**	76.4/93.1	77.0/93.1	76.7/92.9	76.0/91.5
L3	87.9/98.6	77.0/95.9	87.9/98.5	NA	94.4/99.3	**94.6/99.4**	84.6/98.5	84.2/98.1	84.6/97.9	87.5/98.2
M1	94.5/97.4	70.3/79.7	93.9/97.6	NA	86.0/95.3	86.3/95.7	88.7/96.6	**94.5/98.3**	**94.3/97.1**	**94.6/97.9**
M2	80.7/97.3	78.5/96.3	83.8/62.4	82.7/97.3	86.9/97.4	**87.3/97.7**	84.8/98.0	84.3/97.4	84.6/97.7	84.2/96.8
M3	82.3/95.8	69.3/83.4	82.3/95.9	NA	87.6/97.7	87.6/97.6	**89.4/97.7**	81.3/94.5	89.0/97.6	82.5/96.2
S1	57.6/43.9	62.8/62.9	40.4/24.6	40.2/23.3	40.8/23.8	41.1/23.6	**84.9/91.1**	40.2/24.0	40.6/24.4	56.1/53.4
S2	87.2/77.8	77.0/93.7	84.4/98.8	84.8/97.3	**98.1/99.2**	93.5/98.3	87.3/98.5	87.0/97.8	95.8/99.2	87.5/99.0
S3	93.4/97.5	72.7/85.7	85.6/97.5	NA	**94.9/98.3**	94.7/98.9	88.6/98.6	94.1/98.0	88.4/97.8	87.9/97.5
S4	87.5/96.1	78.3/92.0	88.2/96.7	89.7/93.6	**97.0/98.9**	96.8/99.1	88.7/98.0	78.0/86.3	92.8/98.6	78.0/87.1

T1L, type 1 Lang; T2J, type 2 Jones; T3D, type 3 Dearing; T4N, type 4 Ndelle; L, large segment; M, medium segment; S, small segment; NA, not available; Boldface indicates high sequence identity.

### Phylogenetic classification

**Fig 3:**
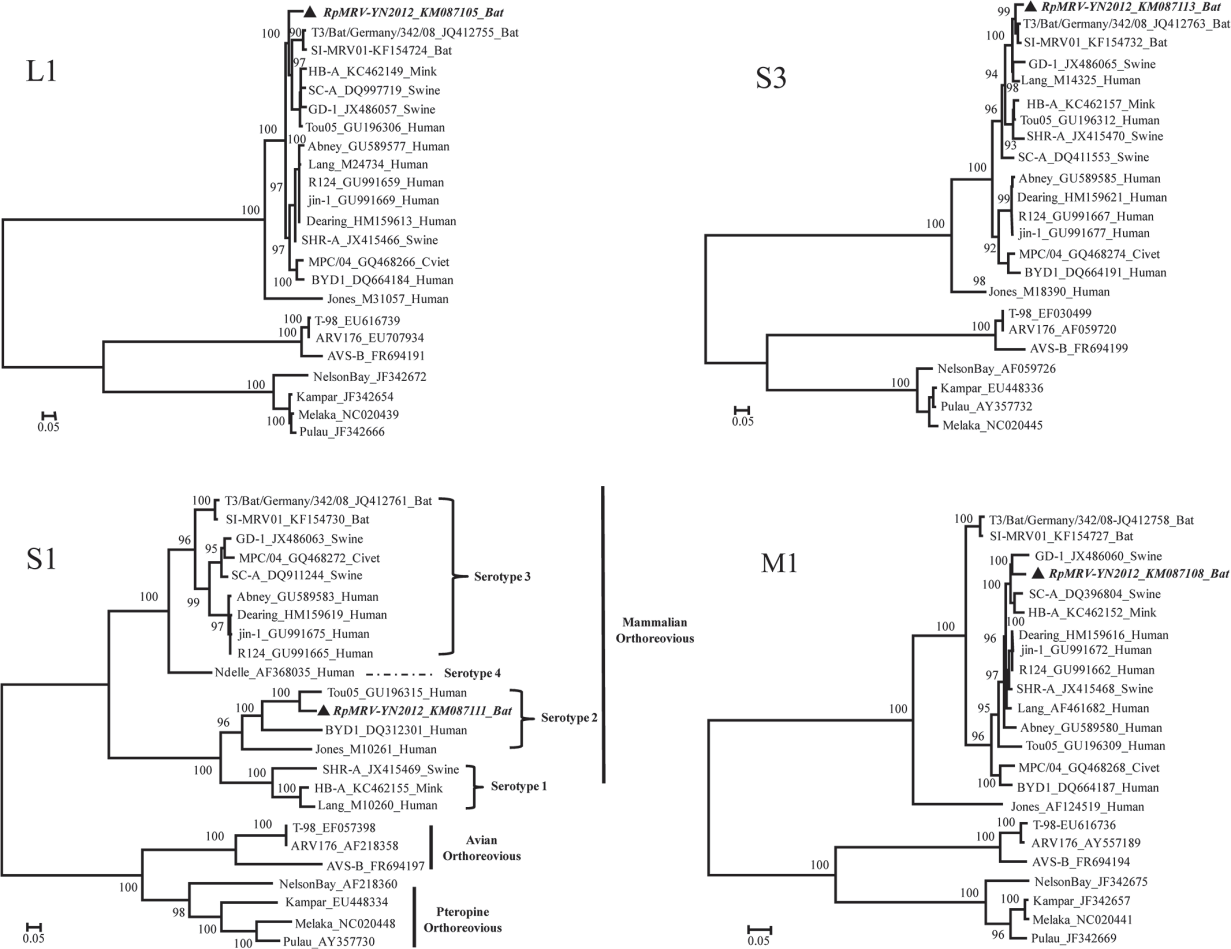
Phylogenetic analysis of the L1, M1, S1, and S3 genome segments for the RpMRV-YN2012 strain and most related whole-genome strains from GenBank. Phylogenetic analyses were performed by the Maximum-likelihood method using MEGA (ver. 6.06, www.megasoftware.net). The scale bar, indicates the number of nucleotide substitutions per site. Bootstrap percentages (1000 replicates) above 50% are shown at nodes.The sequence of RpMRV-YN2012 from this study is in ***bold italic*** typeface.

Whole genome sequences of 10 segments (L1–L3, M1–M3, S1–S4) of RpMRV-YN2012 were aligned with those of *Orthoreovirus* strains available in GenBank. Maximum likelihood trees were reconstructed based on the nucleotide sequence of the whole genome sequences. The topology of the phylogenetic trees confirmed the relationship of RpMRV-YN2012 with these bat, pig, and human MRV strains (Fig. 3). Although all the segments of RpMRV-YN2012 were clustered within the MRVs, they separated in two serotypes (T2 and T3). Commonly, orthoreoviruses are classified taxonomically on the basis of the S1 segment [2]. The topology of a phylogenetic tree based on the nucleotide sequence of the complete S1 segment assigned RpMRV-YN2012 to the human-associated serotype 2 MRVs and was most divergent from the serotype 3 MRVs, providing phylogenetic evidence that RpMRV-YN2012 was a serotype 2 strain (Fig. 3). However, based on the other segments, the reconstructed tree sorted RpMRV-YN2012 to pig- or bat-associated serotype 3 MRVs (Fig. 3).

### Prevalence of RpMRV-YN2012

The RpMRV-YN2012 prevalence study was performed in pharyngeal swabs (*n* = 106) and anal swabs (*n* = 106) of bats (36 *Rhinolophus luctus*, 27 *Rhinolophus affinis*, 25 *Rhinolophus pusillus*, 5 *Penthetor lucasian*, and 13 *Myotis daubentonii*) captured in the six roosts where the urine and feces samples were collected. Total RNA from all collected samples was isolated and screened by a reported RT-PCR procedure, targeting the conserved regions of the L1 genome segment [17]. Four additional anal swab samples from *Rhinolophus pusillus* were found to harbor strain RpMRV-YN2012, whereas other samples *were negative*.

## Discussion

Bats, as the most abundant, diverse, and geographically dispersed vertebrates on earth, have been shown recently to be the reservoir hosts of a variety of zoonotic viruses responsible for severe human disease outbreaks, some with very high mortality [6]. However, reports on the isolation of *orthoreovirus* from bats are limited. Nelson Bay virus (NBV) was the first bat-origin orthoreovirus isolated in 1968 from the heart blood of a flying fox (*Pteropus poliocephalus*) in New South Wales, Australia [22]. In 1999, the second bat-borne orthoreovirus, called Pulau virus (PulV), was isolated from fruit bat urine collected on Tioman Island, Malaysia [23]. In 2007, a novel bat-origin orthoreovirus, Melaka virus (MelV), was isolated from a patient with acute respiratory disease in Melaka, Malaysia, and the data provided suggested that this new orthoreovirus is capable of human-to-human transmission; this was the first report of an orthoreovirus in association with acute human respiratory diseases [24]. In 2008, the fourth member of the NBV species group, Kampar virus (KamV), was isolated from a human patient with fever and acute respiratory illness, and was suggested to be a bat-borne orthoreovirus by epidemiological data [25]. Since then, bat-borne orthoreoviruses have received much attention, and five additional orthoreoviruses (Xi-River, Kampar, Sikamat, HK23629/07 and Broome viruses) have been isolated from fruit bats, or humans with assumed contact with bats [26–29]. MRV infection in humans has been shown to be fairly common and the infections are often shown to be asymptomatic or associated with mild, self-limiting respiratory or gastrointestinal illness in infants and children [3]. Recent studies have shown that MRV can cause severe illnesses in humans and other mammals, including upper respiratory tract infections, encephalitis, and diarrhea[4, 5, 18]. In this study, we describe the first isolation of a novel natural reassortant MRV strain, named RpMRV-YN2012, from the least horseshoe bat (*Rhinolophus pusillus*) during a viral carriage study of bats in southwestern cities of Yunnan Province, China. The results provided evidence that least horseshoe bats carry MRVs and may act as a natural reservoir of MRVs.

The S1 gene segment of mammalian reovirus is bicistronic, encoding both the viral attachment protein sigma-1 (δ1) and the non-structural protein (δ1 s) from overlapping open reading frames [1,3]. The viral δ1 protein is unique to each prototype of mammalian reovirus and determines the serotype and also is the major genetic determinant of neurovirulence in infected mice [1–3]. The S1 gene of RpMRV-YN2012 showed far greater similarity to serotype 2 tou05 (84.9%/91.1%, nt/aa identity) than it did to serotype 1 and 3 MRVs S1 genes (Table 3). The phylogenetic tree based on the nucleotide sequence of the complete S1 segment showed RpMRV-YN2012 closely related to serotype 2 MRVs and was most divergent from the serotype 1 and 3 MRVs. However, based on the other segments, the reconstructed tree sorted RpMRV-YN2012 to serotype 3 MRVs (Fig. 3). These data confirm that RpMRV-YN2012 is a novel serotype 2 MRV that may have originated from reassortment among serotype 2 and 3 MRVs. Until now, most of the orthoreoviruses identified in bats belong to the *Pteropine orthoreovirus* species or serotype 3 MRVs [18, 21, 30]. RpMRV-YN2012 is the first serotype 2 MRV isolated from bats.

Considering RpMRV-YN2012 may have resulted from a reassortment of bat, pig, and/or human MRV strains, which can cause severe human or animal diseases (diarrhea, acute gastroenteritis and necrotizing encephalopathy), it is necessary to identify whether RpMRV-YN2012 is associated with diarrhea, acute gastroenteritis and necrotizing encephalopathy in clinical patients. In addition, we should carefully monitor its evolution and virulence in real time.
